# Development and Preliminary Psychometric Evaluation of the Spiritual Needs Scale for Older Adults Receiving Palliative Care

**DOI:** 10.3390/healthcare14172773

**Published:** 2026-09-01

**Authors:** Linan Cheng, Qian Chen

**Affiliations:** 1 School of Nursing, Wenzhou Medical University, Wenzhou 325035, China; 2 Department of Geriatrics, West China Hospital, West China School of Nursing, Sichuan University, Chengdu 610041, China; chen_qian@scu.edu.cn

**Keywords:** spiritual needs, older adults, palliative care, psychometric evaluation

## Abstract

**Highlights:**

**What are the main findings?**

- The SNS specifically assesses the spiritual needs of older adults receiving palliative care and demonstrated satisfactory validity and reliability.
- The SNS provides a structured approach to identifying unmet spiritual needs among older adults in palliative care.

**What are the implications of the main findings?**

- The SNS may support the integration of spiritual assessment into routine, person-centered, and culturally sensitive palliative nursing care.
- Further external validation is needed to determine the applicability and generalizability of the SNS across different cultural and clinical populations.

**Abstract:**

Background: Meeting the spiritual needs of older adults receiving palliative care requires culturally sensitive and psychometrically sound assessment instruments. This study aimed to develop and evaluate the psychometric properties of the Spiritual Needs Scale (SNS) for older adults receiving palliative care. Methods: The primary version of the SNS was developed through a scoping review, in-depth interviews, Rodgers’ concept analysis, and expert consultation. A consecutive sample of 395 older adults receiving palliative care was recruited from the inpatient wards of two university-affiliated hospitals to evaluate the psychometric properties of the SNS. Validity assessment included content, face, and construct validity, while reliability was evaluated through Cronbach’s alpha and test–retest measures. Additionally, ceiling and floor effects were examined. Results: The SNS consists of 40 items. The scale demonstrated excellent content validity, with an S-CVI/Ave of 0.98 and an S-CVI/UA of 0.77. Exploratory factor analysis (EFA) revealed six factors that explain 65.595% of the total variance, which included connection with oneself and others, transcendence, connection with nature, reflection on life’s meaning, seeking life’s meaning, and a sense of belonging. The overall Cronbach’s alpha coefficient for the scale was 0.963, with coefficients for the six factors ranging from 0.852 to 0.920. The scale’s retest reliability was 0.853. Conclusions: The SNS demonstrated satisfactory validity and reliability for assessing the spiritual needs of older adults receiving palliative care. Further studies are needed to validate the scale in different cultural and clinical populations.

## 1. Introduction

The global population is aging rapidly, leading to a significant increase in spiritual needs among older adults who are seeking meaning and coping mechanisms for age-related challenges [1,2,3,4]. This trend is particularly pronounced in China, which has entered a phase of moderate population aging [5,6]. According to the 2024 National Report on the Development of Aging Undertakings, 310.31 million individuals are aged 60 and above, accounting for 22.0% of the total population, including 220.23 million people aged 65 and older, representing 15.6% of the population [6,7]. Addressing the spiritual needs of this large and expanding demographic has thus become a critical priority for both society and the healthcare system.

Spirituality is increasingly recognized as an essential component of health, especially within the context of palliative care for older adults [8,9]. “Spirituality is a dynamic and intrinsic aspect of humanity through which persons seek ultimate meaning, purpose, and transcendence, and experience relationships with themselves, their family members, communities, society, and nature with the significant or sacred. Spirituality is expressed through beliefs, values, traditions, and practices” [10]. In the context of palliative care, we conceptualize this multidimensional construct as encompassing the pursuit of meaning, purpose, connection, and self-transcendence. For older patients facing serious illness, spirituality serves not only as a psychological adaptive mechanism but also as a vital source of socio-emotional support [7,11,12]. When adequately supported, spiritual well-being can foster resilience, hope, and confidence in confronting health-related challenges and functional decline [3,13,14]. Conversely, unmet spiritual needs may precipitate spiritual distress and compromise overall care outcomes [1,7,13,15]. This understanding has led major healthcare accreditation bodies to mandate spiritual assessments in clinical settings [16,17,18], underscoring its importance in geriatric palliative care, where spiritual considerations are integral to holistic treatment approaches [4,18].

Several instruments have been developed to assess spirituality and spiritual needs; however, they differ considerably in their conceptual focus, target populations, and measured domains. The Functional Assessment of Chronic Illness Therapy–Spiritual Well-Being (FACIT-Sp) primarily evaluates spiritual well-being through the dimensions of meaning, peace, and faith rather than assessing unmet spiritual needs [19]. The Spiritual Needs Assessment for Patients (SNAP), including its Turkish version (T-SNAP), focuses on psychosocial, spiritual, and religious needs among patients with serious illness [20], whereas the Spiritual Care Needs Inventory evaluates patients’ expectations regarding spiritual care provision [21]. The Spiritual Well-Being Scale (SWBS) measures overall spiritual well-being instead of specific spiritual needs [22,23]. In addition, the Spiritual Needs Questionnaire (SNQ), which has been translated and cross-culturally adapted into Chinese, assesses several domains of spiritual needs, including religious, existential, inner peace, and giving/generativity needs [1,7,24]. Although these instruments have demonstrated satisfactory psychometric properties in their original settings, they were developed within different cultural and philosophical contexts and were not specifically designed for older Chinese adults receiving palliative care. Consequently, they may not fully capture culturally embedded spiritual concerns, such as familial harmony, maintaining ancestral connections, establishing a harmonious legacy, and achieving inner peace through non-theistic perspectives.

This limitation is particularly relevant in China, where spirituality is shaped by the long-standing interaction of Confucianism, Buddhism, and Taoism rather than being primarily expressed through organized religion [7]. These traditions emphasize familial responsibility, intergenerational relationships, harmony with nature, and self-cultivation as important sources of meaning in later life. Cockell and McSherry et al. have emphasized that culturally responsive spiritual care requires assessment instruments that adequately reflect these contextual characteristics [1,4,25]. Despite increasing recognition of spiritual care in China, a considerable gap remains between the growing demand for spiritual assessment and the availability of culturally appropriate measurement tools, highlighting the need for an indigenous instrument.

Therefore, this study aimed to develop and psychometrically validate the Spiritual Needs Scale (SNS), a native instrument specifically designed to assess the multidimensional spiritual needs of older adults receiving palliative care in China. Grounded in the lived experiences of the target population and informed by a culturally sensitive and non-theistic philosophical framework, the SNS is intended to complement existing instruments by capturing culturally specific dimensions of spirituality while providing healthcare professionals with a practical tool for individualized spiritual assessment and person-centered palliative care.

## 2. Methods

### 2.1. Development of the SNS

The Spiritual Needs Scale (SNS) was developed by extending the four-step instrument development framework proposed by Waltz et al. [26,27]. To establish a comprehensive and culturally appropriate conceptual foundation, the development process integrated evidence from a scoping review, Rodgers’ evolutionary concept analysis, qualitative interviews, and expert consultation.

First, a published scoping review was conducted to systematically identify existing conceptualizations, assessment domains, and measurement instruments for spiritual needs among older adults, thereby identifying current research gaps and informing the preliminary content domains of the scale [27]. Second, Rodgers’ evolutionary concept analysis was performed to clarify the conceptual attributes, antecedents, and consequences of spiritual needs and to establish the theoretical framework underpinning the instrument. This analysis identified four conceptual dimensions—individual, interpersonal, environmental, and transcendental—which served as the theoretical basis for item development [1]. Third, a descriptive qualitative study using semi-structured interviews was conducted with 36 participants, including older adults, caregivers, and healthcare professionals. Participants were recruited using purposive and theoretical sampling strategies to ensure variation in age, health status, care settings, and caregiving experience. Interview data were analyzed using qualitative content analysis, with independent coding conducted by two researchers followed by consensus discussions to resolve discrepancies. The interview findings were integrated with the evidence from the concept analysis and scoping review to supplement and refine the content of the initial item pool. Finally, the preliminary items were reviewed and refined through expert consultation, and the final item pool was developed using a five-point Likert response format.

### 2.2. Determining Its Psychometric Properties

#### Face and Content Validity Assessments

To check face validity, the SNS was given to 20 older adults receiving palliative care, along with their caregivers, for qualitative feedback on item clarity. Based on their input, revisions were made. In a quantitative evaluation, the same participants rated the importance of each item. For quantitative content validity, the same participants rated each item’s importance on a 5-point Likert scale (1 = ‘not important at all’ to 5 = ‘extremely important’). The impact score (Mean Importance × Frequency [% of respondents rating 4 or 5]) was calculated, and items with a score > 1.5 were retained [28]. Two rounds of expert consultation were conducted to assess qualitative content validity. In the first round, 18 experts participated, followed by 16 in the second round. They offered feedback on the clarity, grammar, wording, scoring, adequacy, and simplicity of the SNS items, which prompted revisions.

Experts were eligible if they (a) worked in one of seven major Chinese regions; (b) had ≥10 years of geriatric nursing experience; (c) had ≥5 years of experience in a hospice care unit; (d) had ≥5 years of experience in management/education; (e) held a bachelor’s degree and an associate senior title or above; and (f) were employed in a general hospital, university, or hospice institution. Recruitment was conducted electronically. Only those who provided informed consent were enrolled; non-respondents after two weeks were excluded.

The demographic characteristics of the experts are described using frequencies and percentages. The expert authority coefficient (Cr) is computed using the expert judgment coefficient (Ca) and expert familiarity coefficient (Cs), where Cr = (Ca + Cs)/2. Cr ≥ 0.7 indicated acceptable reliability; Cr > 0.8 indicated good authority [29].

The mean, standard deviation, coefficient of variation, and expert consensus level were computed for each item. Items were considered to have expert consensus if their mean score exceeded 4.5 (on a scale of 1 to 5), the coefficient of variation was less than 0.25, and the consensus level was at least 70% [30]. Kendall’s coefficient W was used to assess consensus among expert groups [31], with a significance level set at *p* ≤ 0.05.

Quantitative content validity was assessed using the content validity index (CVI) [26], which includes the item-level content validity index (I-CVI) and scale-level content validity index (S-CVI). The S-CVI consists of the universal agreement (S-CVI/UA) and average (S-CVI/Ave) content validity. The I-CVI was calculated by dividing the number of experts who rated an item as 3, 4, or 5 by the total number of experts. The S-CVI/UA was determined as the percentage of items that were unanimously rated 3, 4, or 5 by all experts, whereas the S-CVI/Ave was computed as the average I-CVI across all items.

### 2.3. Construct Validity Assessment and Ceiling and Floor Effects

#### Study Design and Participant

This cross-sectional survey was conducted in geriatric palliative care centers affiliated with two university hospitals in Sichuan Province, China, from March 2021 to March 2022. Inclusion criteria consisted of being aged 60 years or older, receiving palliative medical treatment, possessing the ability to communicate effectively (either without barriers or with corrected vision and hearing), and providing informed consent for voluntary participation in the survey. Exclusion criteria included (1) individuals with traumatic brain injury, diagnosed schizophrenia, severe functional or organic mental disorders, or other psychiatric illnesses and (2) participants who discontinued or withdrew from the study due to severe physical illness symptoms or other reasons.

### 2.4. Data Collection

Hospital management granted approval for the study. Participants were recruited using a consecutive sampling approach. All eligible older adults receiving palliative care during the data collection period were invited to participate. The purpose of the study was explained to potential participants and their caregivers, and written informed consent was obtained from those who agreed to participate. A research team, including departmental head nurses, conducted and supervised the data collection process. A total of 419 participants were recruited. After excluding 24 questionnaires with incomplete responses or failure to meet the eligibility criteria, 395 complete questionnaires were included in the final analysis. All statistical analyses were performed using complete-case data. Contemporary psychometric recommendations suggest that exploratory factor analysis requires an adequate sample size, typically at least 200 participants, together with an appropriate participant-to-item ratio to ensure stable factor solutions [32,33]. Therefore, the final sample of 395 participants was considered adequate for the psychometric evaluation of the SNS.

To assess the test–retest reliability of the measurement tool, an interval of 2–14 days is considered appropriate. Consequently, in this study, a subset of participants was invited to complete the survey for the second time 7 days after their initial completion.

### 2.5. Measures

The self-designed general information assessment tool covers various demographic variables, including gender, age, ethnicity, marital status, religious beliefs, long-term living area, self-rated physical health, chronic illness, emotional state, and negative/adverse events (past 2 years).

The culturally adapted spiritual needs questionnaire (SNQ), suitable for older adults with palliative care [34], consists of five dimensions and a total of 23 items: love and connection, hope and peace, meaning and purpose, relationship with the supernatural, and acceptance of dying. The content validity was 0.980, and the correlation coefficients between each dimension and the total score of the assessment scale ranged from 0.557 to 0.873. The cumulative variance contribution rate of the five common factors was 57.885%. The Cronbach’s alpha coefficient was 0.908, with split-half reliability and test–retest reliability coefficients of 0.926 and 0.902, respectively.

### 2.6. Statistical Analysis

Statistical analyses were performed using IBM SPSS 25.0 for Windows. In total, 395 individuals were included in exploratory factor analysis (EFA).

Item analysis involves assessing item discrimination and item homogeneity. Each item between the top 27% scoring group and the bottom 27% scoring group was analyzed using an independent-samples *t*-test, with *p* < 0.05 indicating good item discrimination. Homogeneity among individual items and the entire assessment tool was examined through correlation analysis. Items with a critical ratio below 3 or a correlation coefficient below 0.3 were eliminated.

EFA was conducted to validate the structural validity. The significance level for statistical tests was *p* ≤ 0.05. Items with factor loadings greater than 0.40 were included.

To assess the reliability of the measurement tool, internal consistency (Cronbach’s alpha) and its retest reliability (Intraclass Correlation Coefficient (ICC)) within groups were calculated. An acceptable threshold for reliability is an effective value of 0.70 [34].

### 2.7. Quality Control

During the survey phase, participants and caregivers received standardized training and survey manuals and were briefed on the research background, objectives, and assessment tool instructions to minimize bias. Any uncertainties were resolved through group discussions, guided by the principal investigator or project advisors.

After completing the questionnaires, two individuals entered the data within 6 h to minimize discrepancies. Data with consistent response patterns and repeated consistent answers were removed. Statistical experts were consulted to select appropriate analysis methods, ensuring accurate data analysis and preventing statistical errors.

### 2.8. Ethical Approval

This study was approved by the Sichuan University Biomedical Research Ethics Committee (ethics number: 2020-697). All the participants provided written informed consent.

## 3. Results

### 3.1. Development of Chinese Version of SNS

The primary version of SNS contained 41 items in the four levels of individual (14 items), interpersonal (12 items), environmental (5 items), and transcendental (10 items) (see Table 1).

### 3.2. Psychometric Evaluation

#### Face and Content Validity Assessments

In the two rounds of expert consultation, the judgement coefficient (Ca) was 0.87 and 0.96; the familiarity coefficient (Cs) was 0.79 and 0.80; and the expert authority coefficient (Cr) was 0.83 and 0.88. Cr ≥ 0.7 is generally considered acceptable for reliability. In this study, the consulting experts demonstrated a relatively high level of authority over these issues, making the results highly reliable (see Table 2).

In the first round of Delphi expert consultation, we modified 28 items, added 12 new items, merged one item, and removed five items. As a result, the number of items in the initial assessment instrument increased from 41 to 47. The modified instrument showed a Kendall’s W consistency coefficient of 0.136 (*p* < 0.001). The S-CVI/UA ratio was 0.41, and the S-CVI/Ave was 0.95.

In the second round of Delphi expert consultation, we received 26 comments from experts regarding the 47 items. Modified items included Item B2, “Need for reconciliation with others (forgiveness and being forgiven).” Therefore, we added four new items: “B2: Hoping for forgiveness from important individuals in life,” “B3: Hoping for reconciliation with those who have harmed you in the past,” “B4: Hoping to let go of past people/events,” and “B5: Engaging in meaningful activities with others (like-minded individuals, those who share interests).” In total, we modified 13 items, added four new items, and removed three items, resulting in a 48-item spiritual needs assessment instrument. Kendall’s W coefficient for the second round of Delphi consultations was 0.171 (*p* < 0.001). The S-CVI/UA was 0.77, and the S-CVI/Ave was 0.98.

In qualitative face validity assessment, none of the items were changed. In quantitative face validity assessment, all items were more than 1.5. At the end of face and content validity assessments, the SNS contained 48 items.

### 3.3. Construct Validity Assessment and Ceiling and Floor Effects

#### 3.3.1. Sample Characteristics

The general demographic characteristics of the participants are presented in Table 3.

#### 3.3.2. Item Analysis

The results of the outlier analysis showed that comparing the high-scoring group (top 27%, total score ≥ 187) with the low-scoring group (bottom 27%, total score ≤ 150), all items had significant *p*-values (*p* < 0.001). Therefore, no items were eliminated owing to poor discriminant validity. In the homogeneity test, items B10 and D9 were eliminated owing to their correlations (R values) being below 0.3 (*p* < 0.001), while the remaining items exhibited correlations above 0.30, indicating good homogeneity of the tool.

#### 3.3.3. Construct Validity

In this study, the Kaiser-Meyer–Olkin (KMO) was 0.941, exceeding the threshold of 0.5. Moreover, Bartlett’s sphericity test yielded a statistically significant result (*X*^2^ = 11,564.347, *p* < 0.001), indicating the suitability of factor analysis for examining the structural validity of the tool. EFA was performed using Principal Axis Factoring (PAF) with Promax oblique rotation. The number of factors was determined based on eigenvalues greater than 1.0, examination of the scree plot, and the interpretability of the factor solution. Items with factor loadings ≥ 0.40 were retained, and the loading on the primary factor was required to be at least twice that on any secondary factor.

**Figure 1:**
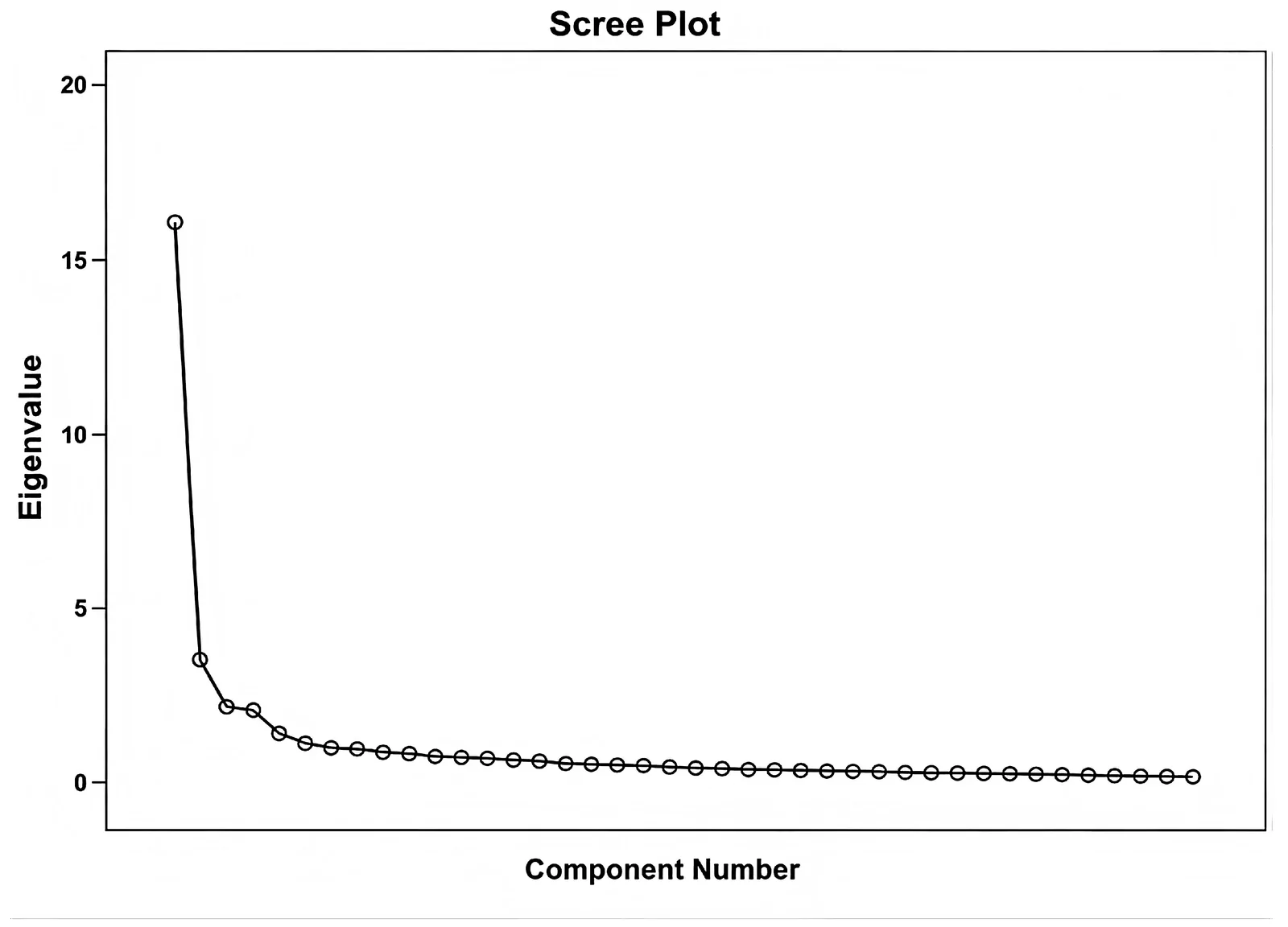
The scree plot of the factor structure of SNS in factor analysis.

Based on the inclusion criteria for the items, the following items were eliminated: B10, B12, B13, D9, D10, B14, B15, and C4. The construct validity of the SNS revealed six distinct factors confirmed by a scree plot (see Figure 1), with eigenvalues greater than 1.0 (16.084, 3.506, 2.149, 2.037, 1.372, and 1.092) and factor loadings ranging from 0.451 to 0.969, all greater than 0.450, on all items (see Table 4).

F1 explained 40.209% of the variance with 17 items and an eigenvalue of 16.084. F2 accounted for 8.765% of the variance with 7 items and an eigenvalue of 3.506. F3 explained 5.372%% of the variance with 5 items and an eigenvalue of 2.149. F4 accounted for 5.092% of the variance with 4 items and an eigenvalue of 2.097. F5 accounted for 3.429% of the variance with 4 items and an eigenvalue of 1.372. F6 accounted for 2.729% of the variance with 3 items and an eigenvalue of 1.092. Together, these six factors accounted for a cumulative variance of 65.595%. All items retained in this model exhibited significant factor loadings above 0.40 on their respective latent variables.

The SNS contained 40 items in the six dimensions of connection with oneself and others (17 items), transcendence (7 items), connection with nature (5 items), reflection on life’s meaning (4 items), seeking life’s meaning (4 items), and sense of belonging (3 items).

#### 3.3.4. Concurrent Validity

Concurrent validity was assessed by examining the relationship between the total score of the new scale and the total score of the Spiritual Needs Questionnaire (SNQ). Since the Shapiro-Wilk test indicated that the data for both scales violated the assumption of bivariate normality, Spearman’s rank correlation coefficient (r) was used. The analysis revealed a strong, statistically significant positive correlation between the two instruments (*r* = 0.778, *p* < 0.01), supporting the criterion validity of the newly developed scale.

#### 3.3.5. Ceiling and Floor Effects

In the assessment of ceiling and floor effects, the relative frequencies of participants with the minimum and the maximum possible total scores of SNS (i.e., 48 and 240) were 10%.

### 3.4. Reliability

The internal consistency of the assessment tool was examined using Cronbach’s alpha coefficient. The overall Cronbach’s alpha coefficient for the SNS was 0.963, and the Cronbach’s alpha coefficients for the seven factors (ranging from F1 to F6) were 0.920, 0.901, 0.865, 0.866, 0.852, and 0.902, respectively. This indicates that the SNS exhibits strong internal consistency.

Twenty-one participants were re-administered to assess the retest reliability of the tool after a 1-week interval. The scores were highly correlated with the initial scores. The overall retest reliability of SNS was 0.853. Therefore, the overall stability of the test–retest reliability is acceptable. However, there is a need to improve the tool further to enhance the stability of each factor.

## 4. Discussion

This study developed the SNS as a culturally sensitive instrument for assessing the spiritual needs of older adults receiving palliative care. Constructed within a non-theistic philosophical framework, the SNS may be applicable not only to Chinese older adults but also to culturally diverse and predominantly secular populations. The scale was developed through a rigorous multi-stage process integrating concept analysis, literature review, semi-structured interviews, expert consultation, and psychometric validation, providing a strong theoretical and methodological foundation. Unlike many existing spiritual needs instruments, the SNS identified six conceptually distinct yet interrelated dimensions: connection with oneself and others, transcendence, connection with nature, reflection on life’s meaning, seeking life’s meaning, and sense of belonging. While transcendence and meaning in life have been reported in previous instruments, the identification of connection with nature, reflection on life’s meaning, and sense of belonging as independent dimensions broadens the current understanding of spiritual needs and highlights aspects that may be particularly relevant to older adults receiving palliative care. Overall, the SNS demonstrated satisfactory psychometric properties and provides a comprehensive framework for individualized spiritual assessment and person-centered spiritual care.

The Delphi consultation process demonstrated strong methodological rigor. The expert panel included specialists from diverse geographical regions and multidisciplinary backgrounds, including clinical nursing, gerontology, philosophy, psychology, medicine, theology, and nursing education, ensuring that the retained items were both theoretically meaningful and clinically relevant. Response rates exceeded 80% in both consultation rounds, surpassing the recommended level for Delphi studies [30] and indicating a high level of expert engagement. The consistently high participation, multidisciplinary consensus, and constructive feedback supported the effectiveness of the Delphi process and reflected the perceived importance of developing a culturally appropriate instrument for assessing spiritual needs among older adults receiving palliative care.

Content validity is fundamental to instrument development because it determines whether the items adequately represent the target construct [27]. In the present study, all items demonstrated excellent content validity, with I-CVI values ranging from 0.88 to 1.00, exceeding the recommended threshold of 0.78 [35], while the S-CVI/UA and S-CVI/Ave further supported excellent overall content validity. After two rounds of Delphi consultation, high expert consensus was achieved, as reflected by the high agreement rate, low coefficients of variation, and significant Kendall’s coefficients of concordance [36]. Furthermore, face validity was strengthened through evaluations by both older adults and caregivers, ensuring that the items were understandable, relevant, and appropriate for the target population [37,38,39]. Together, these findings support the content relevance, clarity, and practical applicability of the SNS.

Exploratory factor analysis supported a six-factor structure of the SNS, comprising connection with oneself and others, transcendence, connection with nature, reflection on life’s meaning, seeking life’s meaning, and a sense of belonging. Together, these factors explained 65.6% of the total variance, indicating satisfactory construct validity [20,37]. The first factor explained 40.2% of the total variance, which may indicate the presence of a relatively strong overarching dimension of spiritual needs while also suggesting a degree of item overlap. This finding should therefore be interpreted with caution, as it may also reflect potential bias in the scale design or item content. However, spiritual needs have been conceptualized as a multidimensional yet integrated construct encompassing individual, interpersonal, environmental, and transcendental domains [1,40,41]. The relatively high variance explained by the first factor may therefore reflect the inherent interconnectedness of these domains rather than inadequate multidimensionality. Consistent with the theoretical framework established through concept analysis, the remaining factors represented conceptually distinct yet interrelated dimensions, supporting the multidimensional structure of the SNS. Nevertheless, further validation using independent samples, confirmatory factor analysis, and bifactor modeling is needed to determine whether a hierarchical factor structure provides a better representation of spiritual needs.

Recent psychometric studies have similarly emphasized the multidimensional nature of spiritual assessment. For example, the newly developed Spiritual Needs in Palliative Care (SNPC) questionnaire identified eight domains encompassing existential, emotional, religious, communication-related, and end-of-life concerns among palliative care patients [40]. Likewise, the I-SPIRIT instrument conceptualized spirituality through three interrelated components—spiritual beliefs, spiritual needs, and spiritual resources—providing a broader assessment framework for patients with serious illness [41]. Although these instruments differ in their conceptual frameworks and target populations, they consistently recognize spirituality as a multidimensional construct extending beyond religious beliefs alone. Unlike the SNPC, which primarily focuses on unmet spiritual needs in palliative care, and the I-SPIRIT, which integrates beliefs, needs, and resources into a comprehensive spiritual assessment, the SNS was specifically developed to assess the multidimensional spiritual needs of older adults receiving palliative care within a culturally sensitive and non-theistic philosophical framework. The inclusion of dimensions such as connection with nature, reflection on life’s meaning, and sense of belonging further reflects the cultural characteristics of spirituality among older adults in the Chinese context.

Given the complexity and cultural context of spiritual needs among older adults, no standardized instrument can comprehensively capture every individual’s spiritual concerns [18,19]. Therefore, the SNS incorporates an open-ended question that allows respondents to describe additional spiritual needs beyond the predefined items. This design complements the quantitative assessment by facilitating the identification of individualized and context-specific concerns that may not be fully reflected in structured questionnaire responses. By combining standardized assessment with individualized inquiry, the SNS provides a practical and flexible approach for comprehensive spiritual needs assessment, supporting person-centered spiritual care across diverse cultural settings.

The absence of ceiling and floor effects further supports the psychometric performance of the SNS. Ceiling and floor effects, commonly defined as occurring when more than 15% of respondents achieve the highest or lowest possible scores, may indicate limited discriminatory ability and reduced measurement sensitivity [1,33,42,43]. In the present study, no ceiling or floor effects were observed, suggesting that the SNS can effectively distinguish different levels of spiritual needs without substantial score clustering at either extreme. This finding supports its suitability for assessing the heterogeneous spiritual needs of older adults receiving palliative care.

Reliability refers to the stability and consistency of a measurement tool, indicating that results should be consistent or stable when the tool is used at different times, by different assessors, or with different assessment methods [33,42,43,44]. The internal consistency analysis using Cronbach’s alpha coefficient showed strong consistency for the overall SNS and its factors, with an overall coefficient of 0.963 and factor coefficients ranging from 0.852 to 0.920. Retest reliability is a form of reliability that measures the consistency of results when the same measurement tool is used again after a certain time interval. If the results of the two measurements are highly correlated, then the measurement tool has high retest reliability, indicating stability of results over time [44]. The retest reliability analysis indicated a high correlation between initial and 1-week interval scores, with an overall coefficient of 0.853, suggesting stability over time.

Beyond its psychometric properties, the SNS may contribute to a broader understanding of spirituality in nursing by conceptualizing spiritual needs as a multidimensional aspect of human experience rather than solely a religious construct. The six dimensions suggest that spiritual needs may be understood as an integrated experience encompassing meaning, connectedness with self and others, transcendence, nature, and belonging, supporting a conceptualization of spirituality that extends beyond religious or faith-based concerns. In this respect, the SNS also differs conceptually from the FACIT-Sp, which primarily assesses spiritual well-being through the domains of meaning, peace, and faith. Rather than assessing an individual’s state of spiritual well-being, the SNS focuses on identifying specific domains in which spiritual support may be needed, thereby potentially providing complementary information for spiritual assessment. The scale may also support future nursing research by enabling standardized assessment and evaluation of spiritual care interventions once its measurement properties have been further established [40]. Clinically, the domain-based structure may help healthcare professionals identify specific areas requiring further exploration and facilitate more individualized spiritual assessment and person-centered care for older adults receiving palliative care [40,41,44]. More broadly, the six-dimensional framework is consistent with the holistic philosophy of nursing, which recognizes that caring extends beyond the management of physical symptoms to include meaning, connectedness, relationships, belonging, and human dignity throughout the illness trajectory. Nevertheless, these theoretical and clinical implications remain preliminary and require further evaluation in independent and culturally diverse populations.

### Limitations

This study has several limitations that should be considered when interpreting the results. First, the reliance on self-report questionnaires may introduce social desirability bias and might not fully capture the nuanced thoughts of participants. While this is a common constraint in survey-based research, it implies that the findings represent participants’ perceived states rather than objectively measured behaviors or emotions. Consequently, the relationships between variables might be influenced by shared method variance. Second, the present study represents a preliminary psychometric evaluation based on a single sample of 395 participants, which was used for item analysis, exploratory factor analysis (EFA), and reliability assessment, potentially increasing the risk of sample-specific findings and overfitting. Without confirmatory factor analysis (CFA) in an independent sample, the identified factor structure remains provisional and requires further confirmation before its stability and generalizability can be established. In addition, internal consistency was assessed using Cronbach’s alpha, while McDonald’s omega was not estimated. Future validation studies should therefore confirm the factor structure in independent samples and incorporate McDonald’s omega as a complementary reliability estimate. Third, although the 40-item SNS provides comprehensive coverage of spiritual needs, its length may increase respondent burden and limit clinical practicality among older adults receiving palliative care. Fourth, participants were recruited consecutively from two tertiary hospitals in Sichuan Province, China, which may limit sample representativeness and generalizability. Although developed within a culturally sensitive and non-theistic philosophical framework, the SNS has only been psychometrically evaluated in Chinese older adults receiving palliative care; therefore, its cross-cultural applicability requires further validation in diverse cultural and healthcare settings.

## 5. Conclusions

In this study, we developed and conducted a preliminary psychometric evaluation of the Spiritual Needs Scale (SNS), a culturally sensitive instrument for assessing the spiritual needs of older adults receiving palliative care. The SNS comprises six conceptually distinct yet interrelated dimensions: connection with oneself and others, transcendence, connection with nature, reflection on life’s meaning, seeking life’s meaning, and a sense of belonging. Constructed within a culturally sensitive and non-theistic philosophical framework, the SNS showed promising preliminary psychometric properties for assessing the multidimensional spiritual needs of this population. By providing a structured framework for identifying unmet spiritual needs, the SNS has the potential to facilitate individualized, person-centered spiritual care and contribute to the integration of spiritual care into holistic nursing practice. However, as participants were recruited from two tertiary hospitals in one Chinese province, the external validity and broader generalizability of the SNS remain limited. Further validation using independent and more diverse samples across different cultural and clinical settings is required before broader clinical application can be established.

## Figures and Tables

**Table 1:** Items of the instrument used to assess the spiritual needs of older adults.

Domain	Item	Item description
F1 Individual Domain	A1	Seeking purpose and meaning in life
	A2	Self-affirmation
	A3	Finding inner peace through coping with adversity, including illness
	A4	Contemplating daily life
	A5	Reflecting on end-of-life experiences
	A6	Sense of belonging within oneself
	A7	Understanding the meaning of suffering
	A8	Awareness of dying and death
	A9	Finding spiritual solace through arts, literature, chanting, etc.
	A10	Positive thoughts and attitudes
	A11	Coming to terms with illness, gaining a belief in coping effectively
	A12	Overcoming feelings of loneliness
	A13	Having expectations or plans for future life
	A14	Achieving a satisfying sense of life’s purpose
	A15	Reconciliation with oneself
F2 Interpersonal Domain	B1	Treating others with kindness
	B2	Hoping for forgiveness from important individuals in life
	B3	Hoping for reconciliation with those who have harmed you in the past
	B4	Hoping to let go of past people/events
	B5	Engaging in meaningful activities with others (like-minded individuals, those who share interests)
	B6	Connecting with family (reuniting, accompanying, staying in touch)
	B7	Sharing life experiences/wisdom with others (family members, loved ones, friends, descendants, etc.)
	B8	Comforting and being comforted by others
	B9	Love and being loved by others
	B10	Discussing possibilities of rebirth, afterlife, or reincarnation with others (like-minded individuals, those interested) regarding matters of the afterlife or cycle of life and death
	B11	Hoping for more attention from those around you
	B12	Hoping to discuss your worries or fears with others (health care professionals, family members, relatives, friends, etc.)
	B13	Saying goodbye to relatives and friends
	B14	How family and friends mourn and find closure after death
	B15	Expressing gratitude towards others or repaying kindness
	B16	Reflecting on one’s life journey and completing unfinished business
F3 Environmental Domain	C1	Connecting with nature through poetry, literature, painting, or other means to experience unity with the universe
	C2	Loving and revering nature
	C3	Getting close to and immersing oneself in the beauty of nature to cleanse the soul
	C4	Living and arranging one’s daily routine in harmony with natural laws, such as adjusting clothing and diet based on environmental changes
	C5	Enjoying the marvel and awe of nature
	C6	Hoping for a pleasant living environment
	C7	Wishing for a favorable healthcare environment
F4 Transcendent Domain	D1	Connection with higher ideologies such as communism
	D2	Connection with a higher existence, such as Buddha, God, Allah, etc.
	D3	Belief in or acceptance of the cycle of life and death
	D4	Hoping for a better afterlife
	D5	Searching for ultimate belongingness
	D6	Returning to the embrace of ancestors
	D7	Participating in cultural customs and traditions, such as ancestor worship or commemorations of significant days
	D8	Sense of achievement beyond suffering
	D9	Arranging one’s own funeral or memorial service before death
	D10	Discussing with relatives the stories or experiences related to spiritual sensations like vivid dreams

**Table 2:** Results of Delphi expert consultation.

First-Round Results				Second-Round Results			
NO	Ca	Cs	Cr	NO	Ca	Cs	Cr
1	0.90	1.00	0.95	1	0.90	1.00	0.95
2	0.90	0.90	0.90	2	0.90	0.90	0.90
3	0.90	1.00	0.95	3	0.90	1.00	0.95
4	0.70	0.90	0.80	4	0.90	1.00	0.95
5	0.70	0.90	0.80	5	0.70	1.00	0.85
6	0.70	0.90	0.80	6	0.70	1.00	0.85
7	0.70	0.80	0.75	7	0.50	0.90	0.70
8	0.70	0.90	0.80	8	0.70	0.90	0.80
9	0.90	1.00	0.95	9	0.90	1.00	0.95
10	0.90	1.00	0.95	10	0.90	1.00	0.95
11	0.70	0.90	0.80	11	0.90	1.00	0.95
12	0.90	1.00	0.95	12	0.90	1.00	0.95
13	0.70	0.70	0.70	13	0.70	0.70	0.70
14	0.90	1.00	0.95	14	0.90	1.00	0.95
15	0.50	1.00	0.75	15	0.50	0.90	0.70
16	0.90	0.80	0.85	16	0.90	1.00	0.95
17	0.90	1.00	0.95				
18	0.70	1.00	0.85				
Total	0.79	0.87	0.83	Total	0.80	0.96	0.88

Note: Ca represents the expert’s judgment on the items. Cs represents the expert’s familiarity with the items. Cr represents the authority coefficient; the authority degree of experts is Cr = (Cs + Ca)/2.

**Table 3:** Demographic characteristics of spiritual needs assessment for older adults.

Items		N (M)	% (SD)
Gender	Male	215	54.4%
	Female	180	45.6%
Age (Years)		73.36	9.58
Marital Status	Single	35	8.9%
	Married	265	67.1%
	Separated/Divorced	14	3.5%
	Widowed	81	20.5%
Ethnicity	Han	365	92.4%
	Ethnic Minority	30	7.6%
Long-term Living Area	Rural	124	31.4%
	Urban	201	50.9%
	Town	70	17.7%
Self-rated Physical Health	Good	47	11.9%
	Fair	249	63.0%
	Poor	99	25.1%
Chronic Illness	Yes	332	84.1%
	No	63	15.9%
Emotional State	Anxiety	3.05	1.29%
	Depression	3.09	1.30%
Medical Insurance Coverage	Full Coverage	43	10.9%
	Partial Coverage	321	81.3%
	Fully Self-funded	31	7.8%
Negative/Adverse Events (past 2 years)	Yes	186	47.1%
	No	209	52.9%
Religious beliefs	Yes	88	22.3%
	No	307	77.7%

**Table 4:** Factor loadings of items of spiritual needs assessment for older adults.

		Sum of Squared Loadings			Rotated Factor Loadings					
		Eigenvalue	Variance%	Cumulative%	F1	F2	F3	F4	F5	F6
F1	B9	16.084	40.209	40.209	0.969					
	B8				0.931					
	B6				0.906					
	B7				0.874					
	B4				0.740					
	B5				0.713					
	B2				0.680					
	A11				0.680					
	B1				0.677					
	A10				0.631					
	B3				0.616					
	A14				0.605					
	A15				0.583					
	B11				0.579					
	A13				0.549					
	A12				0.548					
	A9				0.531					
F2	D4	3.506	8.765	48.973		0.775				
	D5					0.769				
	D3					0.745				
	D2					0.692				
	D7					0.673				
	D6					0.644				
	D8					0.556				
F3	C3	2.149	5.372	54.345			0.905			
	C5						0.856			
	C2						0.839			
	C1						0.734			
	B16						0.451			
F4	A5	2.037	5.092	59.437				0.887		
	A8							0.852		
	A7							0.805		
	A6							0.658		
F5	A1	1.372	3.429	62.866					0.905	
	A2								0.834	
	A4								0.729	
	A3								0.659	
F6	C7	1.092	2.729	65.595						0.807
	C6									0.739
	D1									0.521

## Data Availability

The datasets generated and/or analyzed during the current study are not publicly available due to ethical restrictions and the need to protect participant privacy but are available from the corresponding author upon reasonable request.

## Abbreviations

SNS	Spiritual Needs Scale
CVI	Content Validity Index
I-CVI	Item-level Content Validity Index
S-CVI	Scale-level Content Validity Index
KMO	Kaiser–Meyer–Olkin
EFA	Exploratory Factor Analysis
CFA	Confirmatory Factor Analysis
ICC	Intraclass Correlation Coefficient
SWBS	Spiritual Well-Being Scale
SNQ	Spiritual Needs Questionnaire
T-SNAP	Turkish Version of The Spiritual Needs Assessment for Patients
FACIT-Sp	Functional Assessment of Chronic Illness Therapy–Spiritual Well-Being Scale
